# The practice of Daylight Saving Time in Canada: Its suitability with respect to sleep and circadian rhythms

**DOI:** 10.17269/s41997-024-00870-0

**Published:** 2024-03-01

**Authors:** Joseph De Koninck, Ashley Nixon, Roger Godbout

**Affiliations:** 1 School of Psychology, University of Ottawa, Ottawa, ON Canada; 2 Canadian Sleep Research Consortium, https://www.researchsleep.ca/; 3 Département de Psychiatrie, Université de Montréal, Montréal, QC Canada

**Keywords:** Daylight Saving Time, Sleep, Biological rhythms, Canadian time zones, Public health, Heure avancée, sommeil, rythmes biologiques, fuseaux horaires canadiens, santé publique

## Abstract

Daylight Saving Time (DST) is the practice of setting the clocks one hour forward from Standard Time (ST) in the spring and back again to ST in the fall. This commentary discusses the impact of bi-annual time changes on sleep and circadian rhythms and suggests avenues to minimize negative outcomes on the well-being of Canadian citizens. Ideally, ST should be close to solar time, meaning that daylight is equally distributed before and after noon time, i.e., when the sun is at its highest point in the sky. In Canada, some provinces are proposing to opt out of DST to either return to constant ST throughout the year or to implement permanent DST. National and international associations of clinicians and researchers on sleep and biological rhythms and in health sciences have positioned themselves in favour of permanent ST. In Canada, the Canadian Sleep Society and the Canadian Society for Chronobiology have also issued such a position. This commentary focuses on the implications of previous research findings for sleep and health in Canada given its northern geographical location. It concludes with a research agenda focusing on the Canadian context.

## Résumé

L'heure avancée (HA) consiste à avancer les horloges d’une heure par rapport à l’heure normale (HN) au printemps et à revenir à l’HN à l’automne. Le but de ce commentaire est de traiter de l’impact des changements d’heure semestriels sur le sommeil et les rythmes circadiens et de proposer des moyens d’en minimiser les conséquences négatives sur le bien-être des citoyens canadiens. Idéalement, l’HN devrait être proche de l’heure solaire, ce qui signifie que la lumière du jour est répartie de manière égale avant et après midi, c’est-à-dire lorsque le soleil est à son point culminant dans le ciel. Au Canada, certaines provinces proposent de renoncer à la pratique du changement d’heure pour revenir à une HN constante tout au long de l’année ou mettre en place l’HA de façon permanente. Des associations nationales et internationales de cliniciens et de chercheurs sur le sommeil, les rythmes biologiques et les sciences de la santé se sont prononcées en faveur de l’HN permanente. Au Canada, la Société canadienne du sommeil et la Société canadienne de chronobiologie ont adopté la même position. Le commentaire est centré sur les retombées des résultats de recherches antérieures pour le sommeil et la santé au Canada, compte tenu de sa situation géographique nordique. Il se termine par un programme de recherche axé sur le contexte canadien.

## Introduction

Daylight Saving Time (DST) is the practice of setting the clocks one hour forward from Standard Time (ST) in the spring and back again to ST in the fall. ST is normally close to solar time, meaning that daylight is equally distributed before and after noon time, i.e., when the sun is at its highest point in the sky. DST was widely introduced in Canada in 1914 mainly to save energy. In recent years, many countries have questioned the practice and, in several cases, have contemplated or implemented its abolishment and/or replacement with other time practices. In Canada, some provinces are proposing to opt out of DST to either return to constant ST throughout the year or to implement permanent DST. An important number of national and international associations of researchers on sleep and biological rhythms and in health sciences have produced position statements on the issue (see: https://savestandardtime.com/). In Canada, the Canadian Sleep Society has conducted its own review based on literature up to 2021 (report to CSS by De Koninck et al., 2022), issued its own position (https://css-scs.ca/position-statement-of-the-canadian-sleep-society-on-the-practice-of-daylight-saving-time-dst/), and presented on the topic as part of public lectures for the Canadian Sleep Research Consortium (English: https://www.youtube.com/watch?v=6HzmxdtHK6M; French: https://www.youtube.com/watch?v=tGSBoqBu3ZI). The Canadian Society for Chronobiology has also made its own statement (http://www.chronobiocanada.com/official-statements). Antle (2023) has just published a review which focuses on more recent research. All these documents recommend the termination of the practice of DST in favour of permanent ST. The present commentary focuses on the implication of previous research findings for sleep and health in Canada given its northern geographical location which causes very large variations in natural light exposure between winter and summer. This is exacerbated by the anomalies in the current distribution of time zones with respect to solar time. Finally, we formulate recommendations for specific research focusing on the Canadian context.

## Summary of findings

**Fig. 1:**
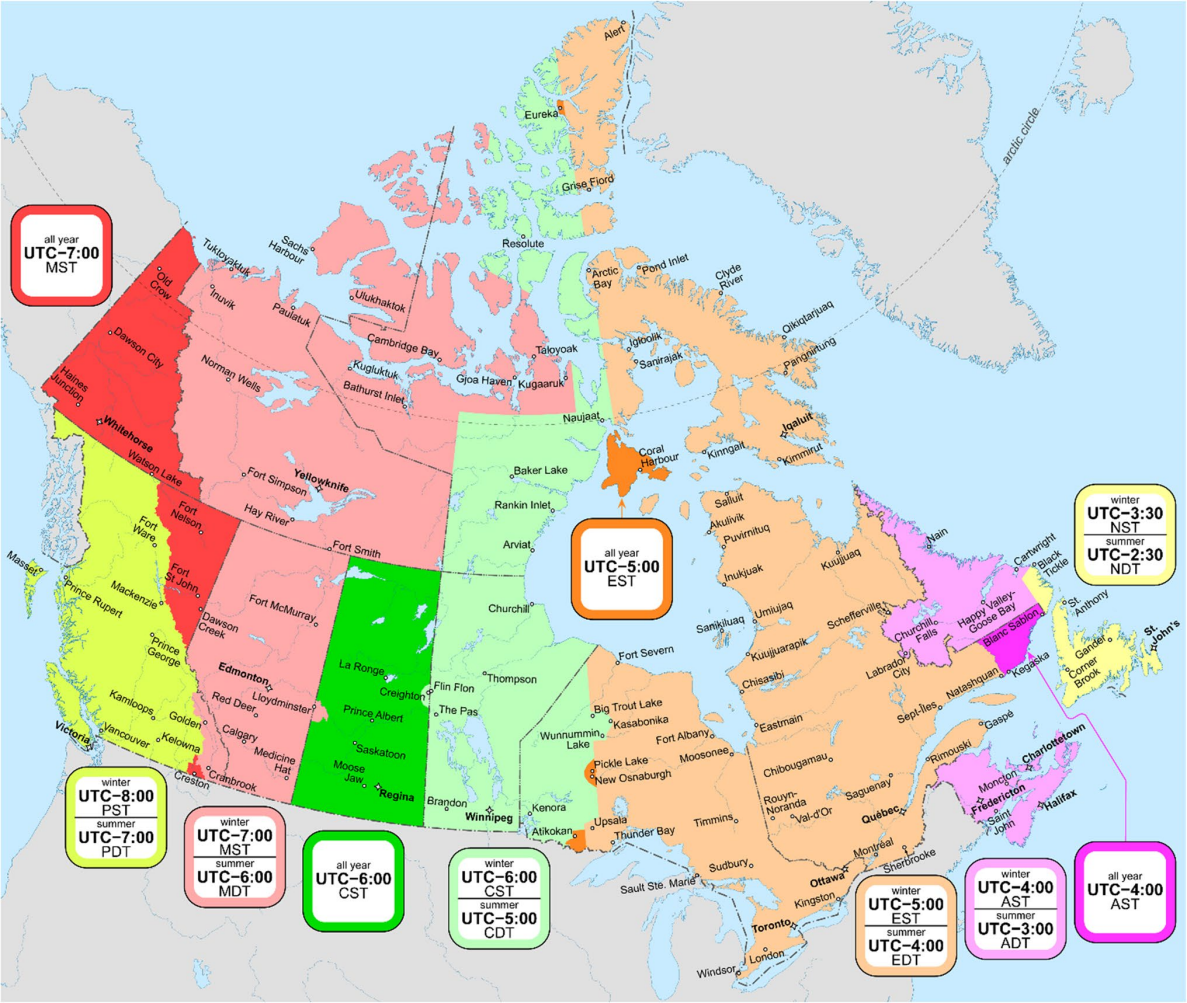
Time zones in Canada. In this updated map, note the very large time zones for Alberta and for Ontario and Quebec. (“Time in Canada”; https://en.wikipedia.org/wiki/Time_in_Canada (Map: MapGrid—Own work, CC BY-SA 4.0, https://commons.wikimedia.org/w/index.php?curid=94413574)

**Fig. 2:**
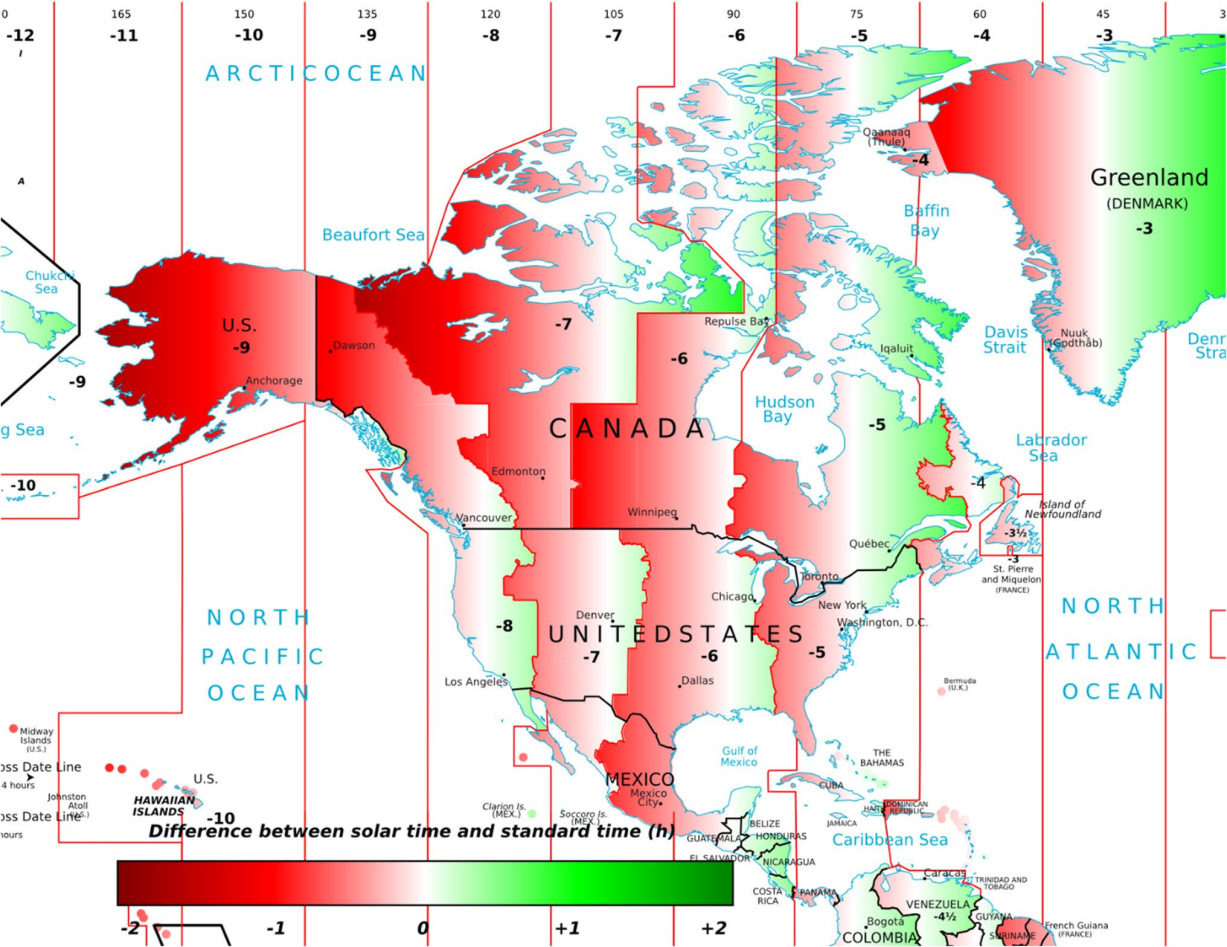
Times zones plus solar time in North America. Regions in red are outside of their solar time zone as a function of colour saturation. This figure is extracted from the world map produced by Stefano Maggiolo who has released his work to the public domain. (https://en.m.wikipedia.org/wiki/File:Solar_time_vs_standard_time.png). Since the map’s drafting in 2015, some Northern regions of Canada have changed their time practice. For example, Yukon and parts of Nunavut are now under permanent ST which improved their time situation

The reviews mentioned above confirm that even if the bi-annual time changes are only of one hour, both induce complaints in the population and psychophysiological disruptions because of sudden misalignments of light exposure and sleep/wake habits governed by the circadian clocks. The March switch to DST is the one that induces the most disruptions since it forces a misalignment of the photoperiod that will continue through the next 8 months. Moreover, the potential one hour loss of sleep can contribute to the immediate negative impact of DST on daytime functioning, physical and mental health issues, as well as reduced overall performances. DST takes place during the night from Saturday to Sunday, giving only one night to adjust sleep schedules before the return to Monday’s school and work activities. It still needs to be documented as to whether implementing DST on Friday night would reduce its immediate impact. As for the misalignment with the biological clock, it enforces later darkness during the summer, favouring delayed bedtime, social jetlag (tendency to move social activities later in the evening), and potentially more sleep loss. This effect is usually reversed with the return to ST in the fall. In the long term, if permanent DST were implemented, it would continue to affect sleep duration because of the permanent misalignment of the biological clock with light exposure. This is confirmed by recent reviews looking at the potential effects of permanent DST on such activities as school performance. Skeldon and Dijk (2019) point out that permanent DST would weaken the beneficial effects of the recent trend to delay school start times, particularly for adolescents, because DST induces social jetlag. At a population level, Roenneberg et al. (2019) compared large populations living in DST versus ST zones or on western versus eastern edges of time zones and found that the advantages of permanent ST outweigh switching to DST annually or permanently. This applies to Canada, which has large time zones that are harmonized with those of the United States at the borders (see Figs. 1 and 2). Time zones and the northern localization of Canada are therefore two important problems arising from this harmonization and are elaborated below.

## The problem with time zones in Canada

Noon is the time when the sun culminates in the sky (i.e., the zenith). Normally, time zones should have been adjusted to solar time so that the sun is at the zenith at noon for people living at the centre of a time zone and the borders of a time zone are plus or minus 30 min away from the centre. Time zones were rather established based on the country’s and provinces’ borders as well as economic activity. As Fig. 1 shows, the Canadian Eastern Time zone is so large that it includes most of Ontario and Quebec so that at each extreme, locations are in the wrong time zone. For example, Thunder Bay, Ontario, is at − 55 min from the centre of the Eastern time zone and Gaspé, Québec, is at + 44 min away from the centre of the same time zone. This means that on December 21, the shortest sun exposure day of the year, sunrise in Thunder Bay is at 08:45 under Standard Time and would be at 09:45 if Daylight Saving Time were applied all year round. In the case of Gaspé, under ST, the sunrise is at 07:08 on December 21 and would thus be at 08:08 under DST but the sunset currently at 15:23 would be at an advantageous 16:23.

Figure 2 superimposes solar time over Standard Time in North America to visualize how large portions of Canada are outside of solar time, particularly towards the west. The red areas are officially in Standard Time but are actually under a DST-type of light exposure schedule. As a consequence, Thunder Bay should theoretically be in the Central Time Zone while Gaspé should be part of the Atlantic Time Zone. Overall, if permanent DST were to be adopted, citizens living in cities more than 30 min behind solar time would experience sunrise later in the winter and “social jetlag”, i.e., daytime activities would be permanently dissociated from optimal light exposure (Antle, 2023; Berument et al., 2010), leading to sleep loss. It is interesting to note that Saskatchewan, which maintains what is officially labelled as Central Standard Time (CST) yearlong, is oddly situated one hour west of the real solar CST. Under permanent DST, citizens of Saskatchewan would thus be seriously affected in winter since noon would be 2 h late relative to solar time. The more natural time of Saskatchewan should be Mountain time zone. This issue of locations on the western portion of time zones and its disadvantages within DST is demonstrated by a recent report by Antle et al. (2022) who analyzed the preferences for DST in the 2021 Alberta referendum depending on the longitudinal (east–west) situation of the voters. The voting choice was between two options: the status quo with a shift to DST in spring versus permanent DST (permanent ST was not an option). Those situated in the western part of the province voted against permanent DST whereas those in the eastern part voted in favour. Clearly, the westerners realized that with permanent DST they would experience exceedingly later sunrise in the winter.

## The problem with the northern localization of Canada with respect to DST

Latitude, i.e., how far one is from the equator, determines the duration of sunlight exposure. For example, close to the equator, duration of daylight and darkness are 12 h each; therefore, switching to DST would have almost no effect on sleep habits. Indeed, Alencar et al. (2017) reported little discomfort with DST in Brazil with no disruption for circadian rhythms. Further north, in Miami, FL, on December 21, the daylight duration is 10:30 h, whereas in Montreal, QC, it is 8:40 h, leaving almost two hours less of light exposure during daytime. This situation leaves little flexibility for shifting time forward as in the case of DST. Indeed, with 8.5 h of illumination, the morning sunrise would be 07:45 (− 4:15 h from zenith) and sunset at 16:15 (+ 4:15 from zenith). If DST were to be implemented in the winter, sunrise would be at 8:45 and sunset at 17:15. As a matter of fact, all major Canadian cities would experience sunrises well after 8 am, and in the case of Calgary and Edmonton, after 09:00. According to Statistics Canada, on workdays, Canadian adults on average go to bed at close to 23:00 everyday and wake up at 6:54 on weekdays while on Sundays they sleep in almost an hour later to 7:50 (Hurst, 2008). Canadian children, students, and workers would have to wake up during darkness and get to school and work without proper daylight exposure. This is what the northern US population experienced in the winter of 1974 when permanent DST was implemented. The measure was abandoned in the fall of that year because of major complaints from the population about morning darkness (see recent report by Beaujon, 2022).

## Conclusions on the Canadian situation

Our conclusions with respect to the Canadian situation align with those of other associations of researchers and clinicians (https://savestandardtime.com/), whereby:

1. Time changes induce problems for Canadian citizens that are comparable to those observed in geographical locations at the same latitude and perhaps more detrimental compared to those in lower latitudes, as documented by Martin-Obella (2019).
2. The ideal practice for better sleep and harmony with the internal biological clock would be permanent ST. In the long term, it should even be more important for the global health of Canadians with a potential reduction in burden of illness.
3. If permanent DST were implemented, most of the Canadian population would be more affected by late sunrise in the morning and sleep deprivation associated with social jetlag than more southern states. This would provoke more issues of mental and physical health.

## Recommendations

We recommend that the Canadian federal and provincial authorities consult researchers, health professionals, and communities before modifying the current time change practices in order that they implement policies that are grounded in science-based well-being. Here is, in order of preference, the proposed course of action:

1. A return to yearlong Standard Time (ST). If implemented, it may be a good opportunity to redefine Canadian time zones so as to optimally fit with better sleep practices. As far as the relationship with our southern neighbours is concerned, it is worth mentioning that Mexico has just approved implementing permanent ST except for cities along its northern border which will harmonize with neighbouring American cities.
2. Meanwhile or if the current practice of bi-annual time changes is maintained, two improvements could be envisaged. First, time changes could be brought closer to the equinoxes, i.e., in early April rather than March (as it was prior to 2006) and in late September instead of November. Sleep and circadian rhythms would then be less disrupted due to the more equal durations of daylight and darkness and it would shorten the length of the excessively long disruptive period of DST. This way, sunrise would occur earlier in the morning and sunset would occur later in the afternoon, possibly reducing the depressive effects of shortened light exposure in the fall. The second improvement would be to implement time changes overnight from Friday to Saturday instead of Saturday to Sunday. This would give more time to adjust sleep schedules before the return to Monday’s school and work activities.

## Future research

While most studies on DST support the above conclusions and recommendations, there are still a number of reports with contradictory findings on the presence of disruptions and/or their severity (Antle, 2023). This seems to be attributable to uncontrolled variables that interfere with the interpretation of results (see below), including longitude and latitude of studied populations and their location within time zones, the actual dates of time changes that vary by country, and the presence of school breaks near time changes. Given the geographical characteristics of Canada, it is surprising that relatively little well-controlled research has been done with respect to the Canadian population following the well-recognized study by Coren in 1996 (Coren, 1996), with the exception of the study by Antle et al. (2022). Based on 1,398,784 traffic accidents across all Canadian provinces in 1991 and 1992 according to the Canadian Department of Transport, Coren observed a significant increase in accidents on the Monday following the spring time change and a decrease following the fall change. Epidemiologic studies using data from Statistics Canada and/or other federal and provincial sources on sleep and health issues could evaluate the presence of previously observed deficits and advantages and determine whether they are present and perhaps amplified due to the Canadian situation with respect to DST. Specifically, health data of populations situated west of their solar time zones could be compared with those populations that are relatively well aligned with solar time. For examples, provincial data from Alberta and Saskatchewan could be compared to British Columbia and New Brunswick, and from cities like Calgary and Regina compared to Vancouver, Ottawa, or Montréal.

It must be remembered that Canada comprises far northern areas where First Nations people and Inuit are living. A better understanding of time practices in such northern areas could increase knowledge on the influence of extreme variations in amount of sunlight exposure across seasons as well as the impact of DST, thus educating policymakers. Prospective studies on the Canadian population are also needed, with measures of sleep and chronobiological variables across the bi-annual time changes using ambulatory monitoring technologies and questionnaires. Such studies should control for age, sex, geographic distribution, and ethnicity to properly evaluate the actual short- and long-term effects of time changes in Canada.

## Data Availability

Not applicable.
